# LPGGNet: Learning from Local–Partition–Global Graph Representations for Motor Imagery EEG Recognition

**DOI:** 10.3390/brainsci15121257

**Published:** 2025-11-23

**Authors:** Nanqing Zhang, Hongcai Jian, Xingchen Li, Guoqian Jiang, Xianlun Tang

**Affiliations:** 1 School of Computer Science and Technology, Chongqing University of Posts and Telecommunications, Chongqing 400065, China; d210201038@stu.cqupt.com (N.Z.); d220201017@stu.cqupt.edu.cn (X.L.); 2 School of Engineering and Technology, Zunyi Normal University, Zunyi 563006, China; 2018030@zync.edu.cn; 3 Chongqing Key Laboratory of Complex Systems and Bionic Control, Chongqing University of Posts and Telecommunications, Chongqing 400065, China; 4 School of Electrical Engineering, Yanshan University, Qinhuangdao 066004, China; jiangguoqian@ysu.edu.cn

**Keywords:** electroencephalography (EEG), partial directed coherence (PDC), gaussian median distance (GMD), graph convolutional networks (GCNs)

## Abstract

**Objectives**: Existing motor imagery electroencephalography (MI-EEG) decoding approaches are constrained by their reliance on sole representations of brain connectivity graphs, insufficient utilization of multi-scale information, and lack of adaptability. **Methods**: To address these constraints, we propose a novel Local–Partition–Global Graph learning Network (LPGGNet). The Local Learning module first constructs functional adjacency matrices using partial directed coherence (PDC), effectively capturing causal dynamic interactions among electrodes. It then employs two layers of temporal convolutions to capture high-level temporal features, followed by Graph Convolutional Networks (GCNs) to capture local topological features. In the Partition Learning module, EEG electrodes are divided into four partitions through a task-driven strategy. For each partition, a novel Gaussian median distance is used to construct adjacency matrices, and Gaussian graph filtering is applied to enhance feature consistency within each partition. After merging the local and partitioned features, the model proceeds to the Global Learning module. In this module, a global adjacency matrix is dynamically computed based on cosine similarity, and residual graph convolutions are then applied to extract highly task-relevant global representations. Finally, two fully connected layers perform the classification. **Results**: Experiments were conducted on both the BCI Competition IV-2a dataset and a laboratory-recorded dataset, achieving classification accuracies of 82.9% and 87.5%, respectively, which surpass several state-of-the-art models. The contribution of each module was further validated through ablation studies. **Conclusions**: This study demonstrates the superiority of integrating multi-view brain connectivities with dynamically constructed graph structures for MI-EEG decoding. Moreover, the proposed model offers a novel and efficient solution for EEG signal decoding.

## 1. Introduction

Brain–computer interfaces (BCIs) refer to systems that directly capture and interpret neural activity to facilitate information exchange between humans and machines [1]. They hold significant application value and promise in the medical field, aiding patients with severe neurological disorders in functional recovery, improved quality of life, and neurological rehabilitation. Representative applications include restoring communication [2], stroke rehabilitation [3], and mental health interventions [4,5].

Electroencephalography (EEG) is broadly adopted in BCI systems [6] because of its non-invasive and high temporal resolution. Among EEG paradigms, motor imagery (MI) can trigger the corresponding neural regions without physical movement. As a result, it is especially suitable for rehabilitation training and prosthetic control in patients with motor impairments. MI-EEG signals mainly show rhythmic activity in the sensorimotor cortex, especially ERD/ERS in the μ- and β bands range (event-related desynchronization/synchronization) [7]. For MI-BCI systems, accurate classification and robust recognition depend on efficient extraction and representation of these features. However, the challenges of precise decoding arise from the inherent low signal-to-noise ratio, non-stationarity, and inter-individual variability of MI signals [8]. We can make improvements in both signal processing and decoding frameworks.

In signal processing, we can enhance signal quality by improving brain connectivity and partition approaches, both of which have been extensively discussed in prior research. For instance, phase-based connectivity measures (e.g., PLV/PLI) overcome artifacts and inter-individual amplitude variations, effectively distinguishing different imagery tasks [9]. Similarly, PDC connectivity reflects regional directionality, outperforming single-channel features and improving classification tasks [10]. Furthermore, fusing connectivity measures, such as spectral coherence, imaginary coherence, and phase difference, enables a comprehensive capture of overall brain network structure [11].

Simultaneously, electrode partitioning based on functional-anatomical correspondence reduces spatial variability and improves the physiological interpretability of EEG features [12]. Regional aggregation not only enhances signal-to-noise ratio but also yields more stable functional connectivity estimates [13]. Partitioning achieved through spectral clustering methods (e.g., K-layer Laplacian averaging or eigenvector averaging) exhibits spatially coherent and distinct lateralization patterns, aligning more closely with the anatomical characteristics of motor imagery tasks [11].

These approaches not only enhance the reliability of EEG feature representation but also lay a solid foundation for constructing brain networks with higher neurophysiological relevance.

In EEG decoding, existing MI-EEG decoding frameworks often rely on combining manually extracted features with shallow classifiers. For instance, discriminative spatial features are commonly extracted using the CSP algorithm and subsequently classified with methods such as LDA [14]. Lee et al. [15] firstly obtains features through CWT and DWT (discrete wavelet transform/continuous wavelet transform), followed by dimensionality reduction via PCA. Subsequently, GMM-UBM (Generalized Universal Background Model) and the EM algorithm are used to optimize the training set. However, it requires pre-setting critical time–frequency parameters, and the final classification is achieved through an SVM. Manual feature design heavily relies on expert prior knowledge and struggles to fully capture the inherent complex nonlinear spatiotemporal structures of EEG signals.

With the development of deep learning, researchers have begun exploring data-driven approaches to automatically learn discriminative features from EEG signals [16,17]. Convolutional Neural Networks (CNNs), leveraging their strengths in spatio-temporal representation, have been widely used in MI-EEG decoding tasks. For example, temporal information has been extensively studied using 1-DCNN [18,19] and multi-scale 1-D CNN [20]. For spatial information, earlier methods typically adopted one of two strategies: applying 1-D CNNs across the sensor channels to capture global spatial patterns [18,19,20], or employing compact 2-D CNN on EEG topography to extract localized spatial features [21,22]. However, these approaches may fail to effectively learn the inherent spatial topological relationships within EEG signals.

Over the past few years, Graph Convolutional Networks (GCNs) have introduced graph-based modeling by embedding spatial topological or functional connectivity among electrodes [23,24]. It is worth noting that establishing appropriate adjacency relationships enables more effective propagation and aggregation of information across the graph structure. This mechanism helps capture interactions between different brain regions and reveal characteristics of the overall network. Compared to CNNs, GCNs offer unique advantages in modeling non-Euclidean spatial data and demonstrate strong potential in MI-EEG decoding tasks.

Despite these advantages, existing GCN methods still face several challenges. Firstly, most models typically rely on static adjacency matrices [25], which cannot adequately represent time varying characteristics of EEG signals. Secondly, single spatial topology approaches (such as geometric distance or functional connectivity) often fail to comprehensively capture the multidimensional interaction patterns between brain regions, limiting feature discriminability [9,26]. Furthermore, many GCNs employ global adjacency modeling [27,28], overlooking task-driven local relationships and failing to capture complex interactions between localized regions. Simultaneously, due to volume conduction effects [29], EEG signals contain overlapping signals from different brain regions, leading to reduce spatial resolution.

To address these challenges, we propose a novel LPGGNet framework. The Local Learning module extracts local temporal and spatial features from EEG signals, while the Partition Learning module captures information within partitions and inter-partitioned features. The Global Learning module extracts global features from EEG signals.

This work offers the following main contributions:

(1) We propose a novel LPGGNet framework with hierarchical architecture designed to capture local, partitioned, and global brain activities in MI-EEG.
(2) A novel partition method for EEG electrodes is introduced to spatially isolate task-relevant brain activities and reduce inter-partition interference.
(3) A novel Gaussian median distance (GMD)-based method is proposed to quantify inter-electrode relationships, which better aligns with the physiological characteristics of EEG signal propagation in the brain.
(4) A BCI-based intelligent wheelchair system is developed to validate the usefulness of the proposed LPGGNet.

The remainder of the paper is arranged as follows: In Section 2, we review related work on feature extraction from EEG data; In Section 3, the proposed methodology is explained; In Section 4, extensive experiments are presented on two datasets; Section 5 provides discussions, and Section 6 concludes the paper.

## 2. Related Works

In BCI studies, extracting features from EEG signals is challenging due to their complex spatial arrangement and non-Euclidean nature. In this section, we provide a comprehensive review of Brain Connectivity and Partitions, CNNs and GCNs in EEG signal decoding. By simultaneously incorporating two complementary brain connectivity approaches, GCNs can better capture spatial representations in EEG recording, as we suggest.

### 2.1. Brain Connectivity and Partitions of EEG Signals

In recent years, extensive research has demonstrated that incorporating brain connectivity and electrode partitioning strategies into EEG analysis can significantly enhance classification performance. Functional connectivity and effective connectivity features between brain regions capture cross-regional interaction information, which is often more discriminative than single-channel features. For example, Leeuwis et al. [30] analyzed multi-scale functional connectivity patterns in motor imagery BCI studies, revealing that connection dynamics at local, large-scale, and global levels are closely correlated with classification performance. Maghsoudi et al. [31] achieved significant accuracy improvements by combining effective connectivity features with hierarchical machine learning methods for hand movement imagery classification.

Furthermore, region-based analysis methods demonstrate strong performance within hemispheric partitioning frameworks. Lun et al. [32] proposed a motor imagery classification method based on left–right EEG differences, validating the advantages of partition strategies for feature discrimination. Zhang et al. [33] examined the effects of electrode density and distribution on motor imagery source localization and classification. The study showed that rational electrode partitioning enhances spatial representation and overall decoding performance.

Due to the scarcity of studies on task-driven partitioning in motor imagery, this paper proposed a four-partition scheme based on four motor imagery tasks. Integrating this partitioning strategy with functional connectivity analysis improves EEG classification performance while enhancing the model’s neurophysiological interpretability.

### 2.2. Convolutional Neural Networks (CNNs)

Due to their end-to-end learning framework and efficient local representation capability, CNNs are extensively employed for modeling the spatiotemporal characteristics of EEG signals. Early studies often reorganized EEG signals into 2D matrices (channel × time) or constructed 3D tensors (e.g., frequency × space × time) via spatial interpolation; these representations were then processed using 2D or 3D convolution operations [34,35]. As an example, Schirrmeister et al. [34] designs an end-to-end CNN architecture that applied 2D convolution to effectively decode original EEG signals. Zhao et al. [35] introduces a novel framework for MI-EEG decoding that leverages a 3D structure and specially designed 3D CNNs to enhance performance.

However, these methods suffer from a fundamental limitation: they rely on discrete convolution defined in Euclidean space, overlooking the intrinsic non-Euclidean spatial relationships and functional connectivity patterns among electrodes [36].

In recent years, the Transformer architecture has also been introduced into EEG signal decoding research, leveraging self-attention mechanisms to capture long-range temporal dependencies and global spatial relationships. Wan et al. [37] proposed EEGformer, utilizing Transformer modules to enhance multi-scale temporal dependency modeling; Liu et al. [38] developed MSVTNet, which fuses multi-scale visual Transformers with EEG features, achieving outstanding performance in motor imagery EEG classification.

These attention-based models offer new direction for EEG decoding while serving as an important complement to traditional CNNs. However, the standard Transformer architecture lacks explicit modeling of electrode spatial topology and functional connectivity, making it difficult to directly capture structural associations between brain regions.

### 2.3. Graph Convolutional Networks (GCNs)

To reduce these limitations of CNNs and Transformer, GCNs have been introduced for EEG signal processing. GCNs can handle non-Euclidean data and explicitly model relationships between electrodes, making them more suitable for representing the brain’s functional connectivity networks [39]. A key aspect of GCNs lies in constructing adjacency relationships between electrodes. Early studies predominantly relied on prior knowledge (e.g., physical distance between electrodes) to build static adjacency matrices. For example, Du et al. [40] used Euclidean distance to construct the adjacency matrix, while Hou et al. [36] utilized the absolute Pearson correlation matrix to build a graph for MI tasks recognition.

Nevertheless, these methods still exhibit notable shortcomings. On one hand, predefined or manually designed adjacency matrices makes it difficult to adapt to individual variability and task-dependent changes [41]. On the other hand, traditional functional connectivity metrics (such as Pearson correlation or phase-locking value) often fail to effectively capture nonlinear or high-frequency dynamic interactions [42].

To overcome these problems, our approach integrates spatial distribution features alongside functional coupling characteristics. Specifically, we introduce a novel adjacency relationship based on Gaussian Median Distance (GMD) to characterize the spatial connectivity between EEG electrodes. This approach better aligns with the physiological characteristics of EEG signal propagation in the brain. Furthermore, to capture directional interactions between signals, we incorporate Partial Directed Coherence (PDC) to reliably obtain causal relationships.

## 3. Materials and Methods

### 3.1. Datasets

Extensive experimental validation on the BCI Competition IV 2a dataset (Dataset A) and a laboratory-collected dataset (Dataset B) was conducted to examine the feasibility of the proposed method. The two datasets are described in Section 3.1.1 and Section 3.1.2.

#### 3.1.1. Dataset A

This dataset originates from the 2008 Brain–Computer Interface Competition [43]. Comprising EEG data from nine subjects, the dataset involves four motor imagery tasks: imagining movements of the left hand, imagining movements of the right hand, imagining movements of the both feet, and imagining movements of the tongue. Each subject participated in two sessions recorded on different days, with the first session designated for training and the second session for testing, constituting a cross-session setup.

**Figure 1:**
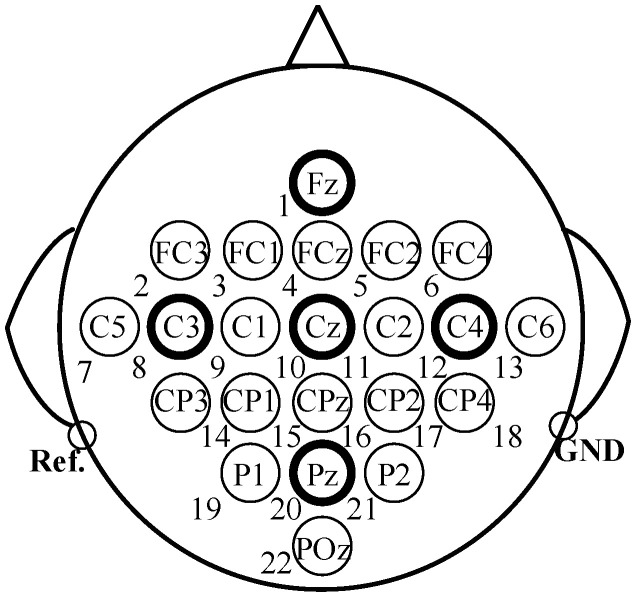
Dataset I Standard 10–20 Electrode Distribution.

Each session contained six runs of 48 trials each, with 12 trials dedicated to each motor imagery task. Thus, each session totals 288 trials. Data acquisition includes 22 EEG channels, with electrode placement as shown in Figure 1.

#### 3.1.2. Dataset B

**Figure 2:**
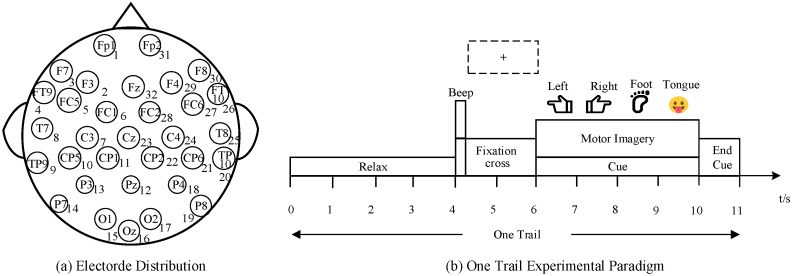
Laboratory Private Dataset. (**a**) Electrode Distribution of Dataset B. (**b**) Experimental Paradigm of Dataset B, illustrating the timing sequence of each motor imagery trial.

This dataset was collected independently by our laboratory and comprises EEG recordings from five subjects. All subjects are healthy, right-handed males aged 22–30. The experiment was approved by the university ethics committee and conducted in a relatively quiet environment. EEG data for each subject is divided into three segments. The experimental paradigm involves four motor imagery tasks: imagining movements of the left hand, right hand, both feet, and tongue. EEG data were recorded with the ActiCHamp system developed by Brain Products GmbH. Thirty-two Ag/AgCl electrodes were used in the system. Fz was the reference electrode, while the other electrodes served to EEG data. Electrode placement is illustrated in Figure 2a.

Each trial lasts 11 s, consisting of four stages: 0–4 s of rest and relaxation, 4–6 s of task instruction, 6–10 s of motor imagery, and a task completion cue at the 11th second (a voice announcement signaling the end of the task). The subsequent trial begins immediately afterward. The temporal sequence of the experimental paradigm is shown in Figure 2b. We collected a total of four sets of motor imagery signals from each subject, with each set comprising 100 trials. Training was performed using the first three sets, and testing with the final set, constituting a Within-Session setup.

#### 3.1.3. Data Processing

Both datasets were collected in an experimental setting. For each trail within each dataset, we acquired 4 s of motor imagery data at a sampling rate of 250 Hz. To address background noise such as electromyography (EMG) and electrooculography (EOG) in the EEG signals, the following measures were implemented: (1) Filtering using a 6th-order Butterworth filter with a bandwidth of 0.5–40 Hz [44]; (2) Removing irrelevant EOG signals; (3) Applying Z-score normalization to reduce sample variability.

### 3.2. LPGGNet

**Figure 3:**
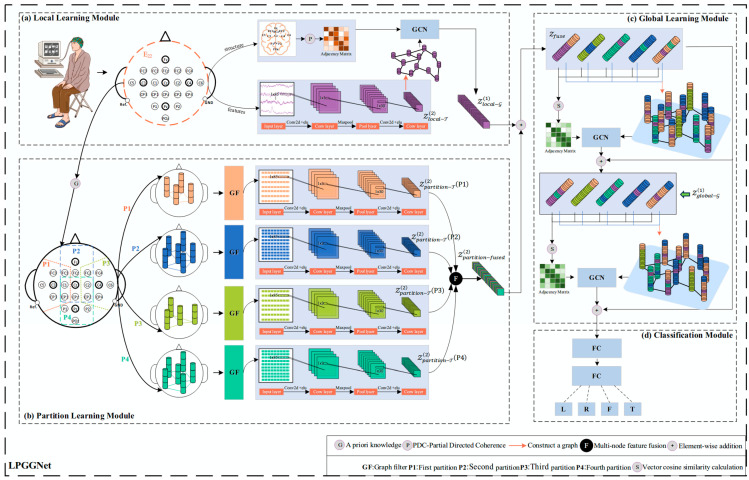
Framework of LPGGNet. (**a**) Learning local features of EEG signals; (**b**) Learning partitioned features of EEG signals; (**c**) Learning global features of EEG signals; (**d**) Classifying EEG signals.

LPGGNet employs a three-layer strategy, with its overall framework illustrated in Figure 3. The specific details are as follows:

(1) Local Learning Module: Adjacency matrices are constructed based on PDC to capture directional dependencies between EEG channels. Following this, two layers of temporal convolutional neural networks (TC) are then applied to capture advanced temporal features from the channel signals. Finally, local topological features of the signals are captured using a graph convolutional network (GCN), which integrates temporal dynamics with their corresponding local graph structures.
(2) Partition Learning Module: An adjacency matrix is first constructed for each partition based on the Gaussian median distance (GMD), upon which a graph filter is built to optimize partition-level signal representations. Subsequently, two layers of temporal CNNs are employed to capture high-level temporal dynamics within each partition. Finally, features from all partitions are integrated using the arithmetic mean method to form a new partition-based representation.
(3) Global Learning Module: Node features obtained from the Local Learning and Partition Learning modules are first fused to unified feature representation. A dynamic global adjacency matrix is then constructed based on cosine similarity to capture inter-node dependencies across all electrodes. To effectively exploit these relationships, two residual graph convolutional layers are employed, which not only extract inter-electrode global features but also alleviate overfitting through residual connections. Finally, the learned global representations are fed into two fully connected (FC) layers to perform classification.

### 3.3. Local Learning Module

To more effectively capture local EEG features, we first apply temporal convolutions to extract their characteristics. Then, PDC are employed to establish relationships between electrodes, enabling directional signal transmission. Finally, a single layer of GCN is applied to propagate EEG features across nodes and extract local topological features.

#### 3.3.1. PDC-Based Electrode Relationships

In GCN-based feature extraction, the adjacency matrix plays a crucial role. Equally important is the direction of information flow, which is essential for analyzing relationships between brain regions. In this study, a partial directed coherence (PDC) algorithm is employed to evaluate the interactions between electrodes. PDC is a widely used frequency domain metric for assessing effective connectivity, derived from the multivariate autoregressive (MVAR) model [45]. It quantifies the directed transmission of data between nodes in multi-channel signals (e.g., EEG) and reveals causal influences at specific frequencies. The core principle of PDC involves frequency domain normalization to eliminate interference from other nodes and highlight directional connections. This approach substantially reduces indirect influences among information streams, emphasizing the most critical relationships of electrodes.

The PDC from node j to node i at frequency *f* can be represented as:

(1) $${PDC}_{ij}\left(f\right)=\frac{\left|A_{ij}(f)\right|}{\sqrt{\sum_{m=1}^{N}{{|A}_{mi}\left(f\right)|}^{2}}}$$

Here, $\left|A_{ij}(f)\right|$ represents the strength of the direct transmission path from node j to node i. $\sqrt{\sum_{m=1}^{N}{{|A}_{mi}\left(f\right)|}^{2}}$ is the total output power range of node *j* to all nodes (including node *i*). ${PDC}_{ij}\left(f\right)\in (0,1)$.

#### 3.3.2. Temporal Graph Convolution Network (TGCN)

EEG signals are multi-channel time series that not only contain rich temporal dynamics but also reflect brain activity recorded across different electrodes, influenced by the spatial topology of the scalp. To effectively capture this inherent spatiotemporal coupling in EEG signals, this paper proposes a TGCN that combines both temporal convolution (TC) and graph convolution (GCN) components, as illustrated in Figure 3a. Firstly, this module performs temporal feature extraction on raw EEG signals using a two TC layers. The core computation of the TC operation can be expressed as:

(2) $$Z_{local-T}^{\left(l+1\right)}={F_{maxpool}(F_{relu}(F}_{bn}(Z_{local-T}^{\left(l\right)}*W_{local-T}^{\left(l+1\right)}+b_{local-T}^{\left(l+1\right)})))$$

Here, $Z_{local-T}^{\left(l\right)}$ is input to the $\left(l+1\right)$th layer. When l=0, $Z_{local-T}^{\left(0\right)}$ represents the preprocessed EEG signal. The symbol T represents Temporal, local refers to the Local Learning Module, and * denotes a 2D convolution (with spatial dimension equal to 1). $W_{local-T}^{\left(l+1\right)}$ represents the convolutional weight, $b_{local-T}^{\left(l+1\right)}$ denotes the bias term, $F_{bn}(\cdot )$ denotes the batch normalization function, $F_{maxpool}$ refers to the max pooling process, and $F_{relu}(\cdot )$ denotes the ReLU nonlinear activation. Specific design is as follows: the first TC layer uses a convolutional kernel of size 1 × 85, aiming to cover a longer time window to capture macroscopic temporal features and reduce computational complexity; the second TC layer uses a smaller convolutional kernel of size 1 × 30 to focus on extracting localized features.

Secondly, the output features $Z_{local-T}^{\left(2\right)}$ are input into the GCN. This layer considers each EEG channel as a node within a graph, where the node features correspond to the temporal representations derived from the TC layer. For simplicity, we define $x={\left(x\left(1\right),x\left(2\right),\ldots ,x\left(N\right)\right)}^{T}=Z_{local-T}^{\left(2\right)}$.

Spectral graph theory forms the basis of GCN. Given an undirected graph G=(V,E) with its adjacency matrix $A\in R^{N\times N}$, degree matrix D=∑A, and identity matrix I. We can define the graph Laplacian operator L:

(3) $$L=I-D^{-\frac{1}{2}}AD^{-\frac{1}{2}}=U\left(\begin{array}{ccc} \lambda_{1} & & \\ & \ddots & \\ & & \lambda_{N} \end{array}\right)U^{T}$$

Here, L was normalized, $U=({u_{1},u}_{2},\ldots ,u_{n})$ denotes the matrix composed of the feature vectors of *L*, $\lambda_{i}$ is feature value of *L*, and N is the number of nodes.

The matrix U encodes the structural information of the graph signal. For classification tasks on graphs, our focus lies in identifying an appropriate convolution kernel g to minimize the classification loss. Thus, the convolution operation, connecting the graph signal with the convolution kernel, is defined as:

(4) $${\left(x*g_{\theta }\right)}_{G}=Ug_{\theta }\left(\Lambda \right)U^{T}x$$

By approximating the convolution kernel using polynomials, we introduce spatial localization. Here, *K* is referred to as the receptive field, meaning that the embedding update of each node involves aggregating embeddings only from its neighbors within *K*-hops. To reduce the computational complexity of convolutions, Chebyshev polynomials are used to approximate the convolution kernel, defined as:

(5) $$g_{\theta }\left(\Lambda \right)\approx \sum_{k=0}^{K}\theta_{k}T_{k}\left(\tilde{\Lambda }\right)=\left\{\begin{array}{ll} T_{0}\left(\tilde{\Lambda }\right)=I & K=0\\ T_{1}\left(\tilde{\Lambda }\right)=\tilde{\Lambda } & K=1\\ 2\tilde{\Lambda }T_{k-1}\left(\tilde{\Lambda }\right)-T_{k-2}\left(\tilde{\Lambda }\right) & K\geq 2 \end{array}\right.$$

Here, $\theta_{k}$ denotes the Chebyshev polynomial coefficient, and $T_{k}(\tilde{\Lambda })$ corresponds to the Chebyshev polynomial with $\tilde{\Lambda }=\frac{2\Lambda }{\lambda_{max}}-I$.

Therefore, Equation (6) represents the convolution operation on graph signals.

(6) $${\left(x*g_{\theta }\right)}_{G}=Ug_{\theta }\left(\Lambda \right)U^{T}x\approx U\left(\sum_{k=0}^{K}\theta_{k}T_{k}\left(\tilde{\Lambda }\right)\right)U^{T}x=\sum_{k=0}^{K}\theta_{k}T_{k}\left(\tilde{L}\right)x\;=\theta x+\theta (D^{-\frac{1}{2}}AD^{-\frac{1}{2}})x\;=\theta (I+D^{-\frac{1}{2}}AD^{-\frac{1}{2}})x\;=\theta {\tilde{D}}^{-\frac{1}{2}}\tilde{A}{\tilde{D}}^{-\frac{1}{2}}x$$

where $\tilde{L}=\frac{2L}{\lambda_{max}}-I$, $\lambda_{max}\approx 2$, K=1, *θ* = *θ*_0_ = *θ*_1_, $\tilde{A}=A+I$, ${\tilde{D}}_{ii}=\sum_{j}{\tilde{A}}_{ij}$.

Thus, the update rule of graph convolution (GCN) is as follows.

(7) $${Z_{G}}^{\left(l\right)}={F_{gelu}(\tilde{D}}^{-\frac{1}{2}}\tilde{A}{\tilde{D}}^{-\frac{1}{2}}X^{\left(l-1\right)}{W_{G}}^{\left(l\right)}+{b_{G}}^{\left(l\right)})$$

Here, at the l-th layer, $Z^{l}$ represents the extracted features, while $W^{l}$ and $b^{l}$ denote the corresponding trainable weight matrix and bias term, respectively. F(·) represents the GeLU activation function.

In this module, the relationship between electrodes is computed via PDC to a directed weighted graph. A single layer of GCN is applied, with input derived from the output $Z_{local-T}^{\left(2\right)}$ of two consecutive temporal convolutions. Therefore:

In TGCN, the adjacency matrix is constructed based on PDC and obtained by averaging all trials for each subject. The input signal is $Z_{local-T}^{\left(2\right)}$, and the output of the TGCN can be expressed as:

(8) $$Z_{local-G}^{\left(1\right)}=F_{gelu}({\overset{ˇ}{A}}_{pdc}Z_{local-T}^{\left(2\right)}{W_{G}}^{\left(1\right)}+{b_{G}}^{\left(1\right)})$$

Here, ${\overset{ˇ}{A}}_{pdc}=\left({{\tilde{D}}_{pdc}}^{-\frac{1}{2}}\right){\tilde{A}}_{pdc}\left({{\tilde{D}}_{pdc}}^{-\frac{1}{2}}\right)$.

### 3.4. Partition Learning Module

This module first adopts a task-driven partitioning strategy to divide EEG electrodes into four partitions. Then, a Gaussian Median Distance-based graph filter is applied to the electrode signals within each partition. Finally, the signals from the four partitions are fused using an arithmetic mean algorithm.

#### 3.4.1. Partition Strategy

Different brain regions are highly interconnected, and different motor imagery (MI) tasks correspond to distinct sensitive areas. Prior knowledge from neuroscience indicates that the four MI tasks have their own sensitive regions. Referencing the key EEG electrodes associated with the four MI tasks—C3 is typically related to left-hand task, C4 to right-hand task [46], CPz and Pz to tongue task [47], and Fz to feet task [48]. In addition, Cz is closely related to all four types of MI.

**Figure 4:**
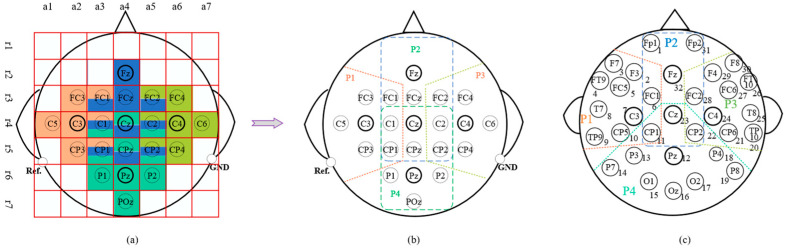
Schematic diagram of electrodes partitioning. (**a**) Electrode color coding, where each color represents a partition, and electrodes with multiple colors indicate that they belong to multiple partitions. (**b**) Electrode partitions in Dataset A. (**c**) Electrode partitions in Dataset B.

Based on this, we divided the 22 electrodes of Dataset A into four partitions, containing 7, 10, 7, and 10 electrodes, respectively, as shown in Figure 4b. Specifically: P1 = {FC3, FC1, C5, C3, C1, CP3, CP1}, P2 = {Fz, FC1, FCz, FC2, C1, Cz, C2, CP1, CPz, CP2}, P3 = {FC2, FC4, C6, C4, C2, CP4, CP2}, and P4 = {POz, P1, Pz, P2, C1, Cz, C2, CP1, CPz, CP2}. As shown in Figure 4a, we use a color coding scheme where the number of colors in a cell corresponds to how many partitions an electrode belongs to. Accordingly, the electrodes shared by three partitions are ${\dddot{\cap }}_{124}=P1\cap P2\cap P4=\{C1,CP1\},{\dddot{\cap }}_{234}=P2\cap P3\cap P4=\{C2,CP2\}$. Those shared by two partitions are ${\ddot{\cap }}_{12}=P1\cap P2=\{FC1\},{\ddot{\cap }}_{23}=P2\cap P3=\{FC2\},{\ddot{\cap }}_{24}=P2\cap P4=\{Cz,CPz\}$.

The foremost principle for defining partition boundaries is the non-overlap of core electrodes. Consider the right-hand MI partition (P1): it must include the C3 electrode. Its right boundary should not extend beyond column a4 (Cz), its upper boundary should not exceed row r2 (Fz), and its lower boundary should not go beyond row r6 (Pz). For the foot area (P2), the left boundary should not exceed column a2 (C3), the right boundary should not exceed column a6 (C4), and the lower boundary should not exceed row r6 (Pz). To ensure symmetry with the tongue area, the lower boundary is designed to include row r5 (CPz). Using the same partitioning method, we divided the 32 electrodes in Dataset B into four partitions. All partitions are shown in Figure 4.

#### 3.4.2. Gaussian Median Distance (GMD) Method

The correlation of EEG electrodes i and j is given by:

(9) $$E[\Phi_{i}\Phi_{j}]=E[\int_{V}G(r_{i},r_{s})I_{m}(r_{s})dr_{s}\cdot \int_{V}G(r_{j},r_{t})I_{m}(r_{t})dr_{t}]$$

where the Green’s function from the source point $r_{s}$ to electrode $r_{i}$ is represented by $G(r_{i},r_{s})$. $\Phi_{i}$ represents the potential recorded by the i-th electrode.

On the scalp surface, the electric potential can be viewed as the projection of the three-dimensional (3D) Green’s function onto the curved scalp geometry. Since the scalp surface is essentially a two-dimensional (2D) manifold, we need to project the 3D Green’s function onto a 2D plane to achieve a 2D approximation. In this study, we approximate the 3D Green’s function as a Gaussian function along the normal direction:

(10) $$G(r,r_{s})\approx g_{0}exp(-\frac{∥r-r_{s}{∥}^{2}}{2l^{2}})$$

Here, l is the correlation length, and $g_{0}$ is a constant.

Normalized electrode correlations:

(11) $$\begin{array}{cc} \rho_{ij} & =\frac{E[\Phi_{i}\Phi_{j}]}{\sqrt{E[\Phi_{i}^{2}]E[\Phi_{j}^{2}]}}=exp(-\frac{d_{ij}^{2}}{4l^{2}}) \end{array}$$

Let $\delta^{2}=4l^{2}$, where *δ* is the median distance. Therefore, the Gaussian median distance of electrodes can be expressed as:

(12) $$G_{ij}\left(d_{ij}\right)=\rho_{ij(d_{ij})}=exp(-\frac{d_{ij}^{2}}{\delta^{2}})$$

From Equation (12), it is evident that the correlation between electrodes decays with the square of their distance, consistent with the propagation pattern of EEG signals within the brain.

In this study, due to the substantial noise present in EEG signals, *δ* is defined as the median of the Euclidean distances between all electrode pairs in each trial to enhance robustness against outliers. $d_{ij}$ denotes the Euclidean distance between electrodes, and is expressed as $d_{ij}=\sqrt{{\left(x_{i}-x_{j}\right)}^{2}+{\left(y_{i}-y_{j}\right)}^{2}+{\left(z_{i}-z_{j}\right)}^{2}}$.

#### 3.4.3. Partitioned Feature Fusion

Partitioned feature fusion is designed to process and integrate features extracted from overlapping electrodes across the four partitions. Each partition calculates the relationship between electrodes using GMD to construct a topological graph. Based on Equation (12), the adjacency matrix for this module is:

(13) $$A_{P}=\left\{\begin{array}{ccc} 0 & if & d_{ij}>\delta \\ \exp\left(-\frac{d_{ij}^{2}}{\delta^{2}}\right) & if & {0<d}_{ij}\leq \delta \\ 1 & if & d_{ij}=0 \end{array}\right.$$

The Gaussian filter is expressed as follows:

(14) $$F={D_{out}}^{-1}L_{P}=I-{D_{out}}^{-1}A_{P}$$

where $L_{P}$ is the Laplace matrix for $A_{P}$. $L_{P}=D_{out}-A_{P}$.

The filtered signal is:

(15) X=FX

Then, X undergoes two consecutive time domain convolutions adopting kernel sizes 1 × 85 and 1 × 30, respectively. The feature extraction is based on Equation (2) but omits the outermost max-pooling operation.

(16) $$Z_{partition-T}^{\left(1\right)}={F_{relu}(F}_{bn}(X*W_{partition-T}^{\left(1\right)}+b_{partition-T}^{\left(1\right)}))$$

(17) $$Z_{partition-T}^{\left(2\right)}={F_{relu}(F}_{bn}(Z_{partition-T}^{\left(1\right)}*W_{partition-T}^{\left(2\right)}+b_{partition-T}^{\left(2\right)}))$$

In this module, the outputs of the four partitions are $Z_{partition-T}^{\left(2\right)}(P1)$, $Z_{partition-T}^{\left(2\right)}$(P2), $Z_{partition-T}^{\left(2\right)}(P3)$, and $Z_{partition-T}^{\left(2\right)}(P4)$, respectively.

As analyzed in Section 3.4.1, the set of electrodes repeated twice is: ${\ddot{\cap }}_{12}\cup {\ddot{\cap }}_{23}\cup {\ddot{\cap }}_{24}=\{FC1,FC2,Cz,CPz\}$, while the set repeated three times is: ${\dddot{\cap }}_{124}\cup {\dddot{\cap }}_{234}=\{C1,C2,CP1,CP2\}$. These electrode features are processed using an arithmetic averaging algorithm to obtain new electrode features, simultaneously eliminating duplicate electrodes. Taking C1 as an example, it belongs to partitions P1, P2 and P4. Its final feature can be expressed as:

(18) $$Z_{partition-T}^{\left(2\right)}\left(C1\right)=\;\Gamma \left(Z_{partition-T}^{\left(2\right)}\left({P1}_{C1}\right),Z_{partition-T}^{\left(2\right)}\left({P2}_{C1}\right),Z_{partition-T}^{\left(2\right)}\left({P4}_{C1}\right);3\right)$$

Here, Γ(features;n) denotes the arithmetic mean of n features.

The partitions after feature processing are labeled as ${P1}_{new}$, ${P2}_{new}$, ${P3}_{new}$, and Therefore, the fused feature can be represented as:

(19) $$\begin{array}{ll} Z_{partition-fused}^{\left(2\right)}=\Pi & (Z_{partition-T}^{\left(2\right)}\left({P1}_{new}\right),Z_{partition-T}^{\left(2\right)}\left({P2}_{new}\right),\\ & Z_{partition-T}^{\left(2\right)}({P3}_{new}),Z_{partition-T}^{\left(2\right)}({P4}_{new});channel) \end{array}$$

Here, Π(features;channel) denotes concatenation along the channel dimension.

### 3.5. Global Learning Module

The global learning module fuses the node features from the Local Learning module with those from the Partition Learning module. It then computes the cosine similarity between electrodes to construct the adjacency matrix of the Global Learning module. Finally, two residual GCNs are applied to extract global spatial features while mitigating the risk of overfitting.

The fused features between nodes are as follows:

(20) $$Z_{fuse}=\Pi \left(Z_{local-G}^{\left(1\right)},Z_{partition-T}^{\left(2\right)};features\right)$$

Here, $\Pi \left(;features\right)$ denotes concatenation along the feature dimension.

The cosine similarity between two vectors is:

(21) $$\cos(\eta_{i},\eta_{j})=\frac{\eta_{i}\cdot \eta_{j}}{∥\eta_{i}∥∥\eta_{j}∥}$$

The adjacency matrix of the global module can be represented as:

(22) $$A_{global}=\left(\begin{array}{ccc} \cos(\eta_{1},\eta_{1}) & \ldots & \cos(\eta_{1},\eta_{N})\\ \vdots & \ddots & \vdots \\ cos(\eta_{N},\eta_{1}) & \ldots & cos(\eta_{N},\eta_{N}) \end{array}\right)$$

Here, N represents the total number of channels, and $\eta_{i}$ denotes the feature of the i-th channel. $A_{global}$ is dynamically updated in each training iteration to enable adaptive learning.

Finally, according to the GCN update Equation (7), the output of the first layer of the dual-layer residual GCN is:

(23) $$Z_{global-G}^{\left(1\right)}=F_{gelu}({\tilde{D}}_{1}^{-\frac{1}{2}}{\tilde{A}}_{1}{\tilde{D}}_{1}^{-\frac{1}{2}}Z_{fuse}{W_{G}}^{\left(1\right)}+{b_{G}}^{\left(1\right)})$$

Here, ${\tilde{A}}_{1}=A_{global-1}+I$ denotes the adjacency matrix for the first-layer of the residual GCNs. The corresponding degree matrix is given by ${\tilde{D}}_{1}=\sum {\tilde{A}}_{1}$.

The second layer of the residual GCNs yields:

(24) $$Z_{global-G}^{\left(2\right)}=F_{gelu}\left({\tilde{D}}_{2}^{-\frac{1}{2}}{\tilde{A}}_{2}{\tilde{D}}_{2}^{-\frac{1}{2}}\left({Z_{fuse}+Z}_{global-G}^{\left(1\right)}\right){W_{G}}^{\left(2\right)}+{b_{G}}^{\left(2\right)}\right)+Z_{fuse}$$

Here, ${\tilde{A}}_{2}=A_{global-2}+I$ denotes the 2nd-layer residual GCN’s adjacency matrix, and ${\tilde{D}}_{2}=\sum {\tilde{A}}_{2}$ denotes the corresponding degree matrix.

### 3.6. Classification Module

The feature $Z_{global-G}^{\left(2\right)}$ is input to two successive FC layers, with the output obtained via a Softmax function. The cross-entropy loss function is used in LPGGNet model.

(25) $$\mathrm{Loss}=-\sum_{i=1}^{C}y_{i}log\left(p_{i}\right)$$

Here, p denotes the predicted probability, C stands for the total classes, y is the ground truth label. Adam optimizer, used in the model, combines the advantages of RMSprop and AdaGrad by incorporating momentum and adaptive learning rates, which helps to accelerate convergence. This makes Adam an efficient approach for updating network weights during training.

## 4. Experiment and Results

### 4.1. Evaluation Metrics

We employ several common metrics to evaluate the proposed model. We first introduce accuracy [49], which represents the proportion of correctly predicted samples. Its calculation is given by Equation (26):

(26) $$Acc=\frac{TP+TN}{TP+FN+FP+TN}$$

Additionally, accuracy of classification is further assessed using the Kappa coefficient [50]. This metric takes into account the possibility of random consistency in the classification results. It is particularly well-suited for evaluating MI-EEG tasks, it reflects the stability and reliability of the model in decoding motor imagery signals. It can be expressed by Equation (27).

(27) $$Kap=\frac{P_{a}-P_{e}}{1-P_{e}}$$

Here, $P_{a}$ denotes the actual observed consistency ratio, while $P_{e}$ represents the expected random consistency ratio.

### 4.2. Model Parameters

All models in this paper were developed using the PyTorch deep learning framework. All experimental procedures were carried out on a desktop computer powered by an NVIDIA GeForce RTX 4070 GPU and an Intel Core™ i7-12700F CPU. The development environment comprised Python 3.8.1, PyTorch 1.10.1, and CUDA 10.2. The hyperparameters were set as follows: learning rate 0.001, dropout 0.5, regularization coefficient 0.069, batch size 64, and weight decay 0.01. The epoch count for Datasets A and B was set to 300, respectively. All these parameters were determined through a grid search during the experiments to ensure optimal model performance on the validation set. To mitigate the impact of randomness during model training, results are presented as the average of one hundred independent experiments.

### 4.3. Experiment on Data A and Data B

#### 4.3.1. Overview Performance

To comprehensively evaluate the proposed model, we benchmarked it against a range of classical and state-of-the-art methods on Dataset A and Dataset B. The compared models spanned from FBCSP (2012) and Shallow/Deep ConvNet (2017) to more recent ones like TS-SEFFNet (2021), Conformer (2022), and GECNN (2024). Table 1 and Table 2 present a summary of the experimental results.

A brief description of the compared models is as follows.

FBCSP [51]: A feature extraction method based on CSP.

Shallow ConvNet [34]: Consisting of two convolutional layers, this compact convolutional neural network is specifically developed for efficient EEG signal classification.

Deep ConvNet [34]: A deep convolutional network structured with five modules, each responsible for distinct stages of spatial and temporal feature processing.

TS-SEFFNet [52]: A time spectrum-based compressed excitation feature fusion network.

SWLDA [53]: SWLDA selects significant features step by step based on statistical criteria to construct an optimal linear classifier.

GAT [54]: GAT introduces a self-attention mechanism to dynamically compute the importance weights of neighboring nodes.

Conformer [55]: A compact convolutional transformer combining the advantages of CNN and Transformer.

GECNN [56]: A graph embedding convolutional neural network.

As shown in Table 1, our model significantly outperforms other comparison methods on Subjects 2, 3, and 6, achieving accuracy rates of 72.9%, 95.5%, and 76.7%, respectively. Regarding the overall metrics of average accuracy (Avg) and Kappa coefficient (Kap), the proposed model achieved the highest values (82.9% and 0.772), indicating superior overall classification performance and consistency. Despite slight underperformance against Conformer or GECNN on specific subjects (e.g., 1, 4, 7, 8), it exhibited exceptional stability across all subjects, confirming its strong generalization and robustness.

As shown in Table 2, our model also performed well, achieving the highest accuracy on four out of five subjects. The Avg reached 87.50%, with a Kap of 0.8392, both significantly outperforming other comparison models.

To demonstrate the statistical significance of the classification accuracy achieved by the proposed model, we employed the nonparametric Wilcoxon signed-rank test [57] on Dataset A. Using the proposed LPGGNet as the baseline, we conducted pairwise comparisons for each reproduced model. The *p*-values from the tests are listed in Table 3. The table shows that the *p*-values for the proposed method versus each reproduced method are all less than 0.05. This indicates that the proposed method achieves significantly higher recognition accuracy compared to other methods.

Combining the experimental results from Table 1, Table 2 and Table 3, our model maintains stable and high performance across different subjects and datasets. The results confirm that the proposed LPGGNet is both effective and advanced for MI-EEG classification.

To further evaluate the stability and distribution characteristics of each model, we plotted violin plots of classification accuracy on Dataset A and Dataset B. These plots integrate the features of box plots and density distributions, providing an intuitive visualization of the model’s central tendency, dispersion, and overall distribution.

**Figure 5:**
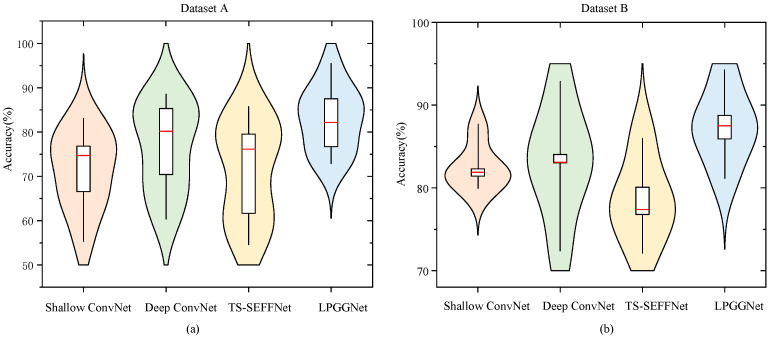
Violin plots of classification accuracy for Datasets A and B. (**a**) Shows the distribution of classification accuracy for four neural network models on Dataset A. (**b**) Shows the distribution of classification accuracy for four neural network models on Dataset B. The red line represents the median accuracy, the white box indicates the interquartile range, and the kernel density shape reflects the overall distribution of accuracy values across subjects.

According to Figure 5, the proposed LPGGNet provides enhanced classification performance and robustness for both datasets. The violin plots show that the median accuracy of LPGGNet is notably higher than that of the comparison models, with a more compact and skewed distribution. This demonstrates that LPGGNet consistently maintains high accuracy with minimal fluctuations across subjects. Compared to the comparison models, LPGGNet not only attains higher average accuracy but also exhibits stronger generalization ability and robustness. Moreover, it effectively alleviates performance variability induced by individual differences, thereby validating the effectiveness of its architecture.

#### 4.3.2. Ablation Experiments

In this section, we conducted ablation experiments at two levels to validate the effectiveness of the proposed LPGGNet. First, we performed ablation experiments on Dataset A and Dataset B for each major module (i.e., local learning, partition learning, and global learning modules) to evaluate their contributions. The experimental results are shown in Table 4.

The findings can be analyzed from three perspectives:

**Single-Module Performance:** When used independently, Partition-only demonstrates the best performance among the three standalone modules, accuracies of 80.1% and 84.92% on both datasets. Such a result significantly outperforms both Local-only and Global-only modules. This indicates that capturing interactions between different partitions is crucial for the MI tasks.

**Module Necessity:** Removing any module from the complete model results in performance degradation, demonstrating that each module is indispensable. Performance degradation was most pronounced when removing the Partition learning module. The complete model achieves 82.9% vs. 73.8% after removal on Dataset A, further validating its critical role. Similarly, removing either the Local Learning or Global Learning module also reduces model performance by 0.6% and 3.5%, respectively, on Dataset A. This indicates all three modules possess irreplaceable functions.

**Synergistic Effect:** The full model (Ours) achieves the best performance on both datasets (82.9% on Dataset A, 87.50% on Dataset B), surpassing all ablated variants. Notably, its performance surpasses the second-best configuration (Local removed) by 0.6% and 0.97% on Datasets A and B, respectively. These findings confirm that the three learning mechanisms are complementary, and their synergistic integration is crucial for achieving optimal and robust classification performance.

In conclusion, the ablation studies solidly verify that each proposed module effectively captures distinct features, and their combination within LPGGNet is both necessary and effective attaining optimal performance.

Second, we conducted further ablation experiments on the components within each major module across Datasets A and B to validate their effectiveness. The results are shown in Table 5.

In the local learning module, replacing the PDC adjacency component with Pearson correlation coefficient led to accuracy decreases of 0.8% and 1.2% on Datasets A and B, respectively. In the partition learning module, replacing the GMD component with an inverse-square component reduced the accuracy by 1.6% and 2.3%, respectively.

Furthermore, when removing the partitioning strategy from the partition learning module, accuracy decreased by 1.2% and 1.9% on Datasets A and B, respectively. When removing the residual connection component from the global learning module, accuracy decreased by 0.6% and 0.5% on Datasets A and B, respectively.

These results indicate that both component replacement and removal lead to model performance degradation, with the most significant impact observed when replacing the GMD component or removing the partitioning strategy. This finding aligns with the conclusions in Table 4, further validating that the partition learning module contributes most significantly to overall performance.

#### 4.3.3. Online Wheelchair Experiment

**Figure 6:**
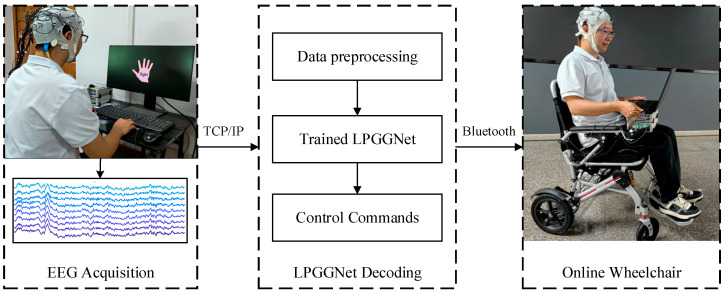
Online Wheelchair Experiment.

To evaluate the applicability of our model in rehabilitation, An intelligent wheelchair control system, grounded in motor imagery BCI, was designed. As shown in Figure 6, it includes signal acquisition, LPGGNet decoding, and wheelchair control. The system first acquires raw EEG signal using BrainAmp equipment from BrainProduct GmbH, Gilching, Germany, and transmits it via TCP/IP. Subsequently, data preprocessing is performed, followed by training using LPGGNet. Finally, the trained outputs are mapped into wheelchair control commands, which facilitates real-time control of the smart wheelchair via Bluetooth.

We invited five subjects from Dataset B to take part in an online experiment. Each subject completed a total of 120 MI tasks across three phases, with 40 tasks per phase. Each task commenced with a clenching signal serving as the starting point for recording MI-EEG signals. Following the clenching signal, subjects maintained a 4 s state of MI. Subsequently, LPGGNet performed decoding and mapped the results to wheelchair commands, with the preprocessing steps following Section 3.1.3 Data Processing. The MI tasks map to four operational commands for the smart wheelchair. Specifically, imagining the left hand corresponds to a left turn, imagining the right hand to a right turn, imagining both feet to forward movement, and imagining the tongue to backward movement.

Table 6 shows the classification accuracy of the online wheelchair experiment. Compared with the offline data listed in Table 2, we observe a lower accuracy in the online experiment due to the more complex environmental conditions during online data collection.

Specifically, challenges in online environments include environmental noise interference, fatigue and attention fluctuations in subjects, system latency, and potential signal loss or anomalies during transmission or processing. To address these issues, future improvement directions may include: (1) optimizing signal preprocessing and filtering methods to enhance noise resistance, (2) designing more robust models to accommodate individual variations and fatigue states, (3) reducing system latency and improving real-time signal processing workflows, and (4) introducing adaptive correction mechanisms to dynamically adjust model performance in online settings.

## 5. Discussion

### 5.1. Effect of Gaussian Median Distance (GMD)

In Partition Learning module, we propose a novel GMD to measure the relationship between electrodes. Compared to traditional methods using an inverse square function of Euclidean distance (ISED) [58] to measure electrode relationships, it effectively suppresses long-range connections, resulting in smoother attenuation. To demonstrate the advantages of GMD, we analyzed data from subject A01 in the BCI IV 2a dataset. Adjacency matrices were constructed using both methods across four partitions, followed by heatmap visualization.

**Figure 7:**
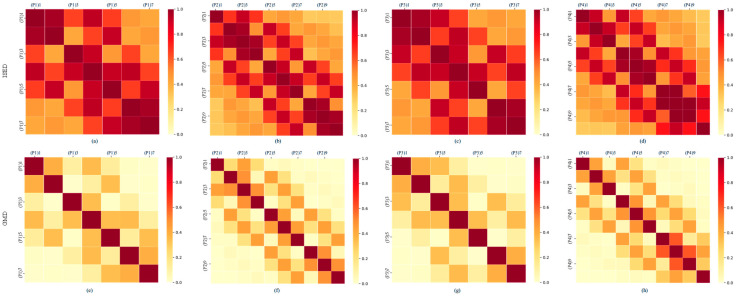
Heatmaps of adjacency matrices constructed based on GMD and ISED. (**a**–**d**) show the adjacency matrices constructed based on ISED for partitions P1–P4, respectively; (**e**–**h**) show the adjacency matrices constructed based on GMD for partitions P1–P4, respectively. (P1) 1 denotes the first electrode in the first partition.

Analysis of Figure 7 reveals that GMD with exponential decay causes weights between long-range electrodes to rapidly approach zero, resulting in a sparser graph structure. In contrast, ISED exhibits slower decay and preserves numerous weak long-range connections, leading to a dense graph structure with poor discriminability. Since connections between long-range nodes persist, ISED often induces a global smoothing effect that introduces noise and weakens local functional relationships. Conversely, GMD emphasizes connections within local neighborhoods, promoting signal smoothing within localized partitions and better preserving the spatial specificity of EEG signals. Overall, the adjacency matrix constructed using GMD not only aligns more closely with the physiological characteristics of EEG signals but also proves more suitable for subsequent graph convolutional learning tasks.

### 5.2. Visualization Analysis of LPGGNet

#### 5.2.1. Visualization of Feature Separation

**Figure 8:**
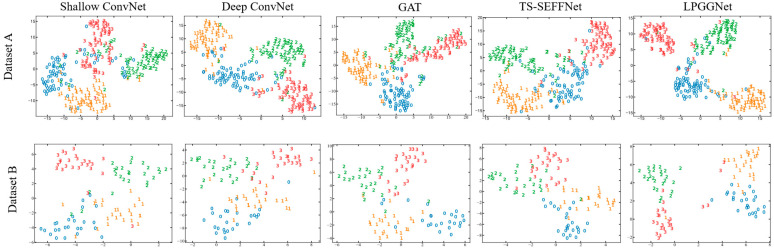
t-SNE visualization of features learned by Subject 3 in Datasets A and B using different methods. The numbers 0, 1, 2, and 3 denotes the category labels corresponding to imagined movements of the left hand, imagined movements of right hand, imagined movements of both feet, and imagined movements of tongue, respectively.

To further investigate the ability of the models to represent EEG features, this paper utilizes the t-SNE [59] algorithm to visualize high-dimensional EEG features learned by the Deep ConvNet, Shallow ConvNet, GAT, TS-SEFFNet, and LPGGNet in a two-dimensional space. As shown in Figure 8, the different models exhibit significant differences in feature separability and clarity of inter-class boundaries.

Specifically, features extracted by Shallow ConvNet and TS-SEFFNet exhibit considerable overlap across the four classes, particularly with highly conflated distributions between Class 2 and Class 3 samples. The fuzzy class boundaries and large distances between samples within the same class indicate feature redundancy and limited discriminative power. Deep ConvNet and GAT demonstrate improved feature clustering, with markedly increased inter-class distances and more compact intra-class samples. However, small transitional regions exist between categories, which indicates that the discriminability of features still needs to be improved.

In contrast, LPGGNet exhibits the clearest feature distribution, forming distinct and stable boundaries between categories. This model significantly enhances feature separability, enabling samples within the same category to achieve higher clustering in low-dimensional space while effectively widening the distance between different categories. This demonstrates that LPGGNet more fully captures task-relevant discriminative information in EEG signals, thereby forming a more structured and recognizable representation in the feature space.

Further analysis from the perspective of category confusion reveals that Shallow ConvNet and GAT models exhibit significant overlap between categories 2 and 3. TS-SEFFNet shows insufficient distinction between categories 0 and 1, while Deep ConvNet improves separation for both pairs of categories. LPGGNet effectively mitigates multi-class confusion, exhibiting only minor overlap between Class 0 and Class 1 while maintaining the clearest overall classification boundaries. This outcome reflects its superiority in capturing distinct activation patterns across brain regions corresponding to different motor imagery tasks.

#### 5.2.2. Visualization of Electrode Contributions

**Figure 9:**
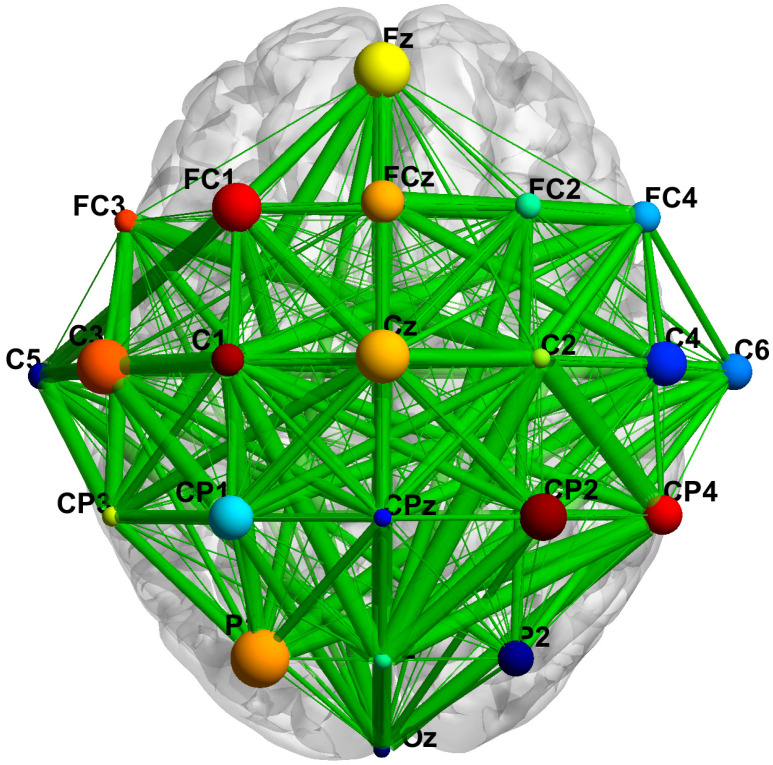
Visualization of electrode contributions for subject 3 in dataset A using BrainNet Viewer. Larger nodes indicate higher electrode contribution during LPGGNet’s decision-making process.

To demonstrate the contribution of electrodes in the LPGGNet decision-making process, we visualized Dataset A using BrainNet Viewer [60] (Figure 9). Node sizes in the figure reflect the magnitude of each electrode’s contribution to the model’s classification. Noticeable differences in node sizes among electrodes indicate uneven contributions to the model’s decision-making.

Specifically, electrodes Fz, FC1, C3, Cz, C4, P1, and Pz exhibit large node sizes, indicating their higher contribution to model classification. Among these, C3, Cz, and C4 are located in the bilateral motor cortex and central parietal regions; Fz and FC1 are in the anterior central region; and P1 and Pz are in the parietal midline region. These brain areas align closely with the neural cortex associated with the motor imagery task. In contrast, electrodes such as FCz, CP1, CP2, CP4, C6, and FC4 exhibit medium node sizes, playing a supplementary role in model classification. Electrodes located in marginal or occipital regions show smaller node sizes, exerting limited influence on model output.

Overall, the spatial distribution of electrodes indicates that LPGGNet primarily relies on signals from central and central-parietal regions for discrimination in motor imagery tasks. This aligns closely with known motor cortex activity, further validating the physiological interpretability of the model’s spatial feature extraction.

### 5.3. Practicality of LPGGNet

To further evaluate the practicality of the proposed model, we compared its parameters, training time, and computational complexity (FLOPs). As shown in Table 7, LPGGNet exhibits a slight increase in parameters (0.513 M) and computational cost (486.2 M) compared to Shallow ConvNet, TS-SEFFNet, and GECNN. However, this moderate computational overhead yields significant improvements in classification performance. Meanwhile, the model’s training time increases only slightly to 14.361 min. Given the corresponding performance gains, this additional overhead is acceptable, indicating that LPGGNet achieves a reasonable balance between accuracy and computational efficiency.

These results demonstrate that the proposed multi-level graph learning and feature fusion mechanism effectively enhances feature representation capabilities. It achieves this improvement without significantly increasing the computational burden. This finding validates the model’s practicality and scalability. However, when deployed on resource-constrained embedded systems or mobile devices, the higher FLOPs may cause inference delays or reduced usability. This issue remains an important aspect for further optimization in future research.

### 5.4. Relationship to Existing Hierarchical GCN and Spatio-Temporal GNN Models

While the proposed LPGGNet is conceptually similar to hierarchical and spatio-temporal graph models like DiffPool, T-GCN and EEGFormer, it distinguishes itself through a unique structural design.

DiffPool [61] achieves hierarchical graph representations through learnable pooling. In contrast, LPGGNet constructs partitioned adjacency based on task-driven partitioning and Gaussian median distance, offering greater neurobiological interpretability. T-GCN [62] combines GCN with GRU to jointly model spatio-temporal dependencies. By comparison, LPGGNet employs a hierarchical decoupling approach. It first extracts local temporal and topological features, and then integrates partitioned and global dependencies. EEGFormer [37] learns multi-layer spatio-temporal relationships through attention mechanisms. In contrast, LPGGNet explicitly constructs a multi-layer graph structure based on PDC, Gaussian distance, and cosine similarity.

Overall, LPGGNet integrates the strengths of these models into a three-stage graph structure learning framework tailored to EEG characteristics.

## 6. Conclusions

A novel LPGGNet is proposed in this paper to enhance the decoding performance of MI-EEG signals by capturing brain task information across different granularities of EEG map data. Within LPGGNet, three hierarchical modules are designed to extract EEG signal features. The Local Learning module extracts local features, the Partition Learning module acquires task-relevant partitioned features, and the Global Learning module learns the overall characteristics of fused features. These three modules effectively improve the distinguishability between different motor tasks and enhance the clarity of distinct brain regions. Although the proposed LPGGNet achieves state-of-the-art performance on both a public dataset and a private dataset, it still has some limitations. The current framework relies on predefined GMD and PDC adjacency matrices, which to some extent restrict its flexibility and adaptability in modeling local node features and complex structures. Future work will focus on: (1) incorporating Transformer mechanisms to better capture inter-partition features and dynamically learn adjacency matrices; (2) exploring adaptive scaling factor in Gaussian Median method from a data-driven perspective; (3) integrating multi-brain-region prior knowledge to improve motor imagery classification accuracy.

## Figures and Tables

**Table 1:** Evaluation Results of Models on Dataset A.

Subject	FBCSP 2012	Shallow ConvNet * 2017	Deep ConvNet * 2017	GAT * 2017	TS- SEFFNet * 2021	SWLDA 2022	Conformer 2022	GECNN 2024	Ours
1	76.00	74.75	80.15	82.36	76.17	71.43	**88.19**	87.90	87.5
2	56.50	55.32	60.39	67.12	54.62	43.87	61.46	67.49	**72.9**
3	81.25	80.14	88.53	90.33	85.76	77.35	93.40	93.41	**95.5**
4	61.00	67.00	70.42	68.33	61.63	59.44	**78.13**	71.49	74.3
5	55.00	66.56	71.33	75.67	64.38	51.74	52.08	**83.70**	82.3
6	45.25	63.12	64.66	62.08	58.75	48.20	65.28	60.93	**76.7**
7	82.75	83.11	85.89	88.01	80.69	69.10	**92.36**	90.61	87.8
8	81.25	75.20	83.16	80.90	78.82	74.48	**88.19**	83.76	81.9
9	70.75	76.82	85.31	82.31	79.55	83.97	**88.89**	84.85	87.2
Avg ± Std (%)	67.75 ± 13.73	71.35 ± 8.93	76.64 ± 10.2	77.45 ± 9.79	71.15 ± 11.31	64.40 ± 14.11	78.66 ± 15.3	80.46 ± 11.16	**82.9** ± 7.38
Kap (%)	57.00	61.88	68.56	69.66	62.12	53.00	71.55	74.00	**77.2**

* indicates that the model was reproduced, and the bold font indicates the highest average accuracy.

**Table 2:** Evaluation Results of Models on Dataset B.

Subject	Shallow ConvNet * 2017	Deep ConvNet * 2017	GAT * 2017	TS-SEFFNet * 2021	Ours
1	81.42	83.09	85.34	77.38	**87.50**
2	79.55	83.02	78.83	72.13	**85.91**
3	87.10	92.54	89.05	86.26	**94.05**
4	**82.30**	72.74	82.01	76.79	81.33
5	81.89	84.04	83.55	80.08	**88.75**
Avg ± Std (%)	82.45 ± 2.8	83.08 ± 7.02	83.75 ± 3.8	78.52 ± 8.93	**87.50** ± 4.61
Kap (%)	76.85	78.47	79.02	71.05	**83.92**

* indicates that the model was reproduced, and the bold font indicates the highest average accuracy.

**Table 3:** The *p*-value between our proposed model and other models.

Method	Shallow ConvNet	Deep ConvNet	GAT	TS- SEFFNet	GECNN
*p*-value	0.008	0.015	0.032	0.046	0.020

**Table 4:** Ablation Experiments on Modules of LPGGNet.

Module	Local Learning	Partition Learning	Global Learning	Dataset A	Dataset B
Local only	●	○	○	74.3	80.02
Partition only	○	●	○	80.1	84.92
Global only	○	○	●	70.6	77.00
Local removed	○	●	●	82.3	86.53
Partition removed	●	○	●	73.8	77.18
Global removed	●	●	○	79.4	83.88
Ours	●	●	●	82.9	87.50

● indicates that the module is used, while ○ indicates that the module is removed.

**Table 5:** Ablation Experiments on Components of LPGGNet.

Module	Local Learning		Partition Learning			Global Learning	Dataset A	Dataset B
Component	PDC	Pearson	GMD	Inverse Square	Partition	Residual Links	ACC (%)	ACC (%)
**Method**	○	●	●	○	●	●	82.1	86.3
	●	○	○	●	●	●	81.3	85.2
	●	○	●	○	●	○	82.3	87.0
	●	○	●	○	○	●	81.7	85.6
	●	○	●	○	●	●	82.9	87.5

● indicates that the module is used, while ○ indicates that the module is removed.

**Table 6:** Online Control Performance of Intelligent Wheelchair Using LPGGNet.

Subject	Left	Right	Foot	Tongue	Mean
1	70.66	68.66	88.30	70.12	74.43
2	66.71	61.36	70.56	89.33	71.99
3	73.23	80.00	81.45	63.33	74.50
4	70.12	66.15	86.44	65.00	71.92
5	68.08	71.45	80.10	83.88	75.87
Mean	69.76	69.52	81.37	74.33	73.74

**Table 7:** Comparison of parameters and computational cost on Dataset A.

Models	Parameters (M)	Training Time (min)	FLOPs (M)	ACC (%)
* Shallow ConvNet [26]	0.048	9.836	48.3	71.35
* TS-SEFFNet [41]	0.283	10.210	301.2	71.15
* GECNN [43]	0.193	11.053	263.12	78.83
Proposed	0.513	14.361	486.2	82.90

M: million, min:minutes, ms:millisecond. * indicates that the model was reproduced.

## Data Availability

A publicly available dataset (Dataset A) was used in this study. These data can be found here: https://www.bbci.de/competition/download/competition_iv/BCICIV_2a_gdf.zip (accessed on 20 March 2025). A private dataset (Dataset B) was used in this study. The dataset presented in this article are not readily available because the data are part of an ongoing study. Requests to access the datasets should be directed to corresponding author.
